# Clinical Utility of an Ex Vivo Functional Test in Personalized Cancer Treatment

**DOI:** 10.3390/jpm16060298

**Published:** 2026-05-31

**Authors:** Vered Bar, Adi Zundelevich, Nancy Gavert, Sara Aharon, Bassima Ibrahim, Anna Kosenko, Guy Neev, Ronen Viner, Ravid Straussman, Raanan Berger, Seth J. Salpeter

**Affiliations:** 1 Curesponse, Ltd., Rehovot 7670101, Israel; vered@curesponse.tech (V.B.); adi@curesponse.tech (A.Z.); sara@curesponse.tech (S.A.); bassima@curesponse.tech (B.I.); anna@curespone.tech (A.K.); guy@curesponse.tech (G.N.); rviner@curesponse.tech (R.V.); raanan.berger@sheba.health.gov.il (R.B.); 2 Department of Molecular Cell Biology, The Weizmann Institute of Science, Rehovot 7610001, Israel; nancy.gavert@weizmann.ac.il (N.G.); ravidst@weizmann.ac.il (R.S.); 3 Department of Oncology, Sheba Medical Center, Ramat Gan 5265601, Israel

**Keywords:** functional cancer diagnostic, personalized medicine, in vitro diagnostic, molecular pathology

## Abstract

**Background/Objectives:** Providing optimized and accurate treatment to cancer patients remains a major challenge in oncology care. The emergence of precision medicine tools to match the correct therapy to the patient has significantly advanced treatment modalities in the last few years. While genomics has been shown to be critical in selecting targeted therapies for a specific somatic mutation, the overall clinical benefit of broad genomic sequencing has been found lacking. Here, we evaluate the utility of our previously clinically validated ex vivo functional assay across different treatment scenarios, demonstrating its ability to transform predicted non-responders into predicted responders, rule out ineffective treatments, provide multiple treatment options, and validate physician choices. **Methods:** The evaluation was performed on a post-market surveillance study analyzing 312 patients, from which 278 patients had successful test reports (an 89.1% test success rate), with clinical outcomes available from 45 of those patients. **Results:** We show that in the group of patients with clinical response data, the tests yield a PPV of 91.18% and NPV of 90.91% with clinical utility impacting physician decision in 51.1% of cases. Further analysis of the entire cohort showed the potential of clinical utility to reach up to 59.3% on a large group of patients. **Conclusions:** The accurate prediction of patient response using the test suggests the potential for the platform to improve patient treatment in clinical practice by reducing ineffective drug use and optimizing personalized patient drug regiments.

## 1. Introduction

Personalized approaches for the treatment of cancer are increasingly accessible, leveraging advanced technologies such as genetic profiling and molecular diagnostics [1]. However, these approaches do not offer the majority of patients the ability to assess multiple treatment options simultaneously and do not predict patient response to treatment in a way that will lead to a robust increase in patient response and survival [2,3]. For patients unresponsive to initial lines of therapy, subsequent treatment options are pursued, yet their effectiveness remains uncertain using today’s most advanced technologies [4].

To improve the selection of advanced treatments in cancer medicine, various methods have assessed the predictive ability of functional assays [5]. These methods include dissociating the tumor and testing drugs on patient cancer cells or growing patient cancer avatars in patient-derived xenograft (PDX) mouse models [6,7]. Recently, several groups have explored the use of organoids as a functional assay to model patient drug response. However, these systems do not consider the complex role of the tumor microenvironment in regulating therapy response. Multiple stromal components, such as immune cells, fibroblasts, blood vessels, and even bacteria, have been shown to influence tumor response to treatment, indicating the need to include them in functional predictive assays [8].

To overcome these challenges, ex vivo organ culture systems have been established to maintain the architecture and function of the cancer microenvironment. In particular, the cResponse platform is an innovative functional tool designed to prioritize treatment options, assess their effectiveness, and predict patient clinical response to various therapeutic agents [9]. By utilizing ex vivo culture of fresh tumor tissue, the platform preserves critical biological properties of the tumor and maintains the morphological principal architecture [10,11]. Multiple different cancer therapeutics are tested on the cancer tissue ex vivo for up to one week, enabling evaluation of drug efficacy based on tissue response. This approach has proven to accurately predict patients’ response to different therapeutic modalities [12]. The test’s result, the cScore, combines metrics such as cancer cell death, surviving cell damage, and additional factors to compute the final score to support oncologists in making evidence-based treatment decisions [12]. Notably, in a previous multicenter prospective observational trial, cResponse has demonstrated high predictive accuracy, with a specificity of 77.7% and a sensitivity of 96% across numerous solid tumor types [12].

Here, we explore the value of cResponse in clinical practice by analyzing a large cohort of patient test results and a sub-cohort of those correlated to real-world clinical data. Specifically, our analysis focuses on cResponse’s utility and capacity to identify effective treatment options that complement or surpass initial physician recommendations, towards improving patient response outcomes. By analyzing real-world clinical data, this study seeks to highlight the practical benefits of integrating cResponse into the treatment decision-making process.

## 2. Materials and Methods

### 2.1. Patients

Patient characteristics can be found in Supplementary Table S1. Data were collected from 312 patients tested between 4 February 2021 and 5 August 2024 in Israel, where the test has been certified for commercialization and routine clinical use. Thirty-four samples were excluded due to either the lack of ability to evaluate the physician’s first choice or cases where the only available score was for the physician’s first choice. This resulted in 278 samples being analyzed. All patients signed informed consent and agreed to have their data shared for the purposes of scientific study. Clinical outcomes from patient treatments were calculated based on patient reporting of radiological evaluation or biochemical markers that was used to continue or discontinue their treatment on a specific therapy. All patients were sent a post market surveillance survey that was provided 6 months following the test, to which 45 patients responded with their treatment and response as confirmed by their oncologist.

### 2.2. Ex Vivo Tissue

Fresh tissue is collected from patients either during surgery or by core-needle biopsy and transferred in ice cold medium. An ex vivo organ culture was performed as previously described. Briefly, the tissue is sliced using a tissue slicer to 250 uM slices and cultured at 80% O_2_, 5% CO_2_ for 5 days. Each drug or drug combination is tested in duplicate or triplicate slices, including DMSO control. After 5 days the tissue is collected, fixed in 4% paraformaldehyde and paraffinized for FFPE blocks. The FFPE samples are cut to 5 uM slices, stained with hematoxylin and eosin and examined by a pathologist to determine the response to therapy. A cScore is assigned to each tissue based upon the cancer cell viability, overall tissue integrity, and cancer cell proliferation. A score of 45 is considered effective treatment, as proven previously in our clinical trials (Golan, et al. 2023) [12]. A higher score is expected to translate to a better clinical response to treatment.

### 2.3. Evaluation of Treatment

Tissue immunohistochemistry was performed on 4 μm sections from the FFPE tissue samples. Hematoxylin and eosin (H&E) staining was performed using an automated stainer (Leica Biosystems, Nussloch Germany). Ki67 staining (Thermo Fischer, Waltham, MA USA Antibody (RM-9106); 1:500 dilution) was performed using an automated stainer (BOND RX, Leica Biosystems, Nussloch Germany). Each sample was evaluated by two independent pathologists, and if there was a disagreement higher than 20%, a third pathologist was consulted. The pathologists were shown the tissue fixed immediately after it was resected from the patient and sliced (time 0) and an untreated tissue obtained after 5 days (control) as reference samples. All other treated EVOC samples were evaluated blindly. The pathologists assessed the live or dead viability (Vi) of tumor cells on a scale of 0–100% (compared to the immediately fixed and control samples). The quality of remaining cancer cell health was measured on a scale of 0–4 (QT) by evaluating the nuclear and cytoplasm architecture, cell organization and intercellular adhesion integrity, with 0 representing complete health and 4 representing high damage. Ki67 proliferation (K) factored as a percentage of replicating cells from 0–100%. To account for tissue heterogeneity, the scores are an average of the treated tissue with a particular drug from 3 different tumors. Tissue immunohistochemistry was performed on 4 μm sections from the FFPE tissue samples. Hematoxylin and eosin (H&E) staining was performed using an automated stainer (Leica Biosystems). Ki67 staining (Thermo Fischer Antibody (RM-9106); 1:500 dilution) was performed using an automated stainer (BOND) with slices taken from the first third, middle third and final third of the biopsied specimen. A final score on a scale of 0 (denoted by a) –100 (denoted by c), accounting for all parameters, was obtained using Equation (1):

(1) $$\sum_{a}^{c}\Delta Vi_{T_{0}}^{V^{100}}*X+QT_{0}^{4}*Y+K_{0}^{100}*Z$$

A score of 0 represents completely viable cancer cells, suggesting no response, and a score of 100 represents no viable cancer cells, suggesting complete response. The weighted output of the evaluation was based on coefficients X, Y, Z of cancer viability, cell replication, and cell death marker, corresponding to 0.7, 0.2, and 0.1, respectively. A threshold score of 45 was used to differentiate between non-responders and responders. Both the equation weighting and the threshold score were determined in a previous clinical study where the test outputs were optimized based on the patient response to treatment [12].

### 2.4. Statistics

All data were recorded and analyzed using Graphpad Prism (Version 10.1.2). Chi-square statistical significance was done with a 2 × 2 contingency table between the outcome results from the response data reported by the patients and the hypothetical outcomes predicted by the cResponse test.

## 3. Results

As part of post-market surveillance, data analysis was performed on 312 patient cases, from which 278 patient cResponse test results were provided between February 2021 and August 2024. The 34 (12.2%) tests that did not yield a report were mostly the result of non-viable cancer tissue being provided in the biopsy, which was consistent with the failure rate of general cancer biopsies that do not yield analyzable tissue and require repeat testing [13].

Prior to performing the cResponse tests, oncologists were asked for their intent-to-treat therapeutic, followed by the next four drugs or drug combinations that they were most likely to use. The patient cancer sample was evaluated in the functional assay starting with the intent-to-treat drug first and then the additional listed drugs in order of preference, based on the amount of patient tissue available for evaluation. Results describing drug response and ranking the most efficacious treatments by cScore were then returned to the treating oncologist who applied the results at their discretion.

The 278 patients with results to their cResponse tests received a post-market surveillance survey to report on their clinical response. Forty-five patients responded, and their results were then assessed for correlation with their treatment score on the cResponse report. These patients had various cancer types, and 91.1% (41/45) received second-line treatment or later, with 53.3% of patients receiving third-line therapy or later (24/45) (see Supplementary Table S1), demonstrating a population weighted towards advanced lines of treatment. The cancer type composition of the 45 responding patients paralleled the larger group of 278 with 42% breast cancer (33.8% in the larger group), 9% colon cancer (10% in the larger group), 8.8% pancreatic cancer (5.4% in the larger group) and 8.8% lung cancer (7.55% in the larger group). Thirty-two patients reported response to therapy (either stable disease or partial response within three to six months of performing the test), while 13 patients did not respond to therapy (progressed). Using the previously determined cResponse threshold of 45 and over (on a scale of 0 to 100) to identify treatment efficacy, we found 31 out of 32 drug sensitive patients were correctly identified as responders (96.87% sensitivity CI 0.83–992), and 10 out of 13 of the drug refractory patients were identified as non-responders (76.92% specificity CI 0.46–0.94). The positive predictive value was 91.18% (31/34 CI 0.79–0.96), and the negative predictive value was 90.91% (10/11 CI 0.58–0.98) (Table 1).

Next, we evaluated the clinical utility of the test in these 45 patients by determining the number of instances where the oncologist changed their intent-to-treat therapeutic selection based on the cResponse results. This occurred when cResponse predicted an improved response with an alternate drug from their list, and the intent-to-treat drug was shown to yield no response by the cResponse test. Twenty-nine patients (64.4%, 29/45) received cResponse reports recommending a drug that demonstrated superior efficacy compared to the physician-selected intent-to-treat drug, which was predicted to be ineffective in the cResponse assay (Supplementary Table S2). The oncologist then chose the cResponse-recommended drug in 23 of those cases (clinical utility on the whole cohort: 51.1%, 23/45), and among those, 21 patients showed a positive response to therapy in the clinic (positive predictive value: 91.3%, 21/23).

**Figure 1:**
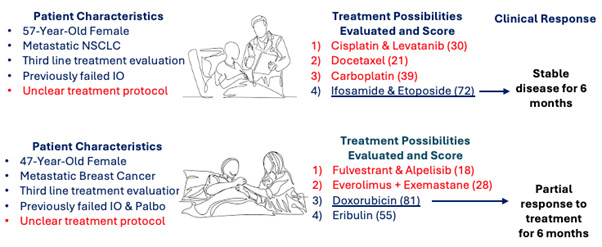
Case studies demonstrating applicability of the cResponse Test. Application of the test was found to identify particular treatments for patients when the physicians first choice was ruled ineffective. Here, are two examples where cResponse identified alternative treatments that yielded efficacy in clinical practice. Red colored treatments were predicted as non-efficacious by the cResponse test (under a score of 45), while the blue scores were predicted as efficacious (over a score of 45).

The impact of these results can be further understood by individual case studies demonstrating the clinical value (Figure 1). A metastatic third-line 57-year-old female with lung cancer was previously treated with various immunotherapies and chemotherapies and had progressed on treatment. Based on the cResponse test results, the patient was treated with the fourth option on the oncologist selection list, Ifosfamide combined with Etoposide, instead of the physician’s planned Cisplatin combined with Lenvatinib regimen. The patient remained with stable disease on treatment for six months. An additional example is a 47-year-old female with metastatic breast cancer, previously treated with immunotherapy followed by Palbociclib, who progressed on treatment. A biopsy from a neck lesion was tested, and based on the results, the planned treatment protocol was switched from Fulvestrant combined with Alpelisib to Doxorubicin. In this case, the patient showed partial response to therapy for 6 months of follow-up.

To gain further understanding of the cResponse test potential clinical application and utility, we analyzed the results of all 278 tests comprised of various cancer types (Supplementary Table S3), dividing the results into four categories: Group 1, where the physicians choice is the only effective treatment found in the test, Group 2, where the physician’s choice and additional treatments are equally as effective based on the test score, Group 3, where all options tested are ineffective based on the test, and Group 4, where the physician’s first choice was predicted to be ineffective (score under 45), and an alternative treatment was found effective based on the test score (as shown in clinical practice in the above examples).

Of a total of 278 successfully tests, at least one effective treatment (groups 1, 2, and 4) was found for 233 patients (83.7%) (Table 2). Group 1 patients made up 6.1% of samples, and group 2 patients made up 18.3%. While these cases did not yield improvement in patient response (since the oncologist selected drug was shown by the test to be effective), doctors noted that these outcomes were also beneficial, as they gave additional confidence in their treatment choice, and when more drug options were identified, it allowed for additional treatment selection that had lower toxicity or cost. Group 3, showing no effective therapy, composed 16.1% of cases, which was also valuable to clinical practice, as it suggested that the physician needed to examine new options for the patient’s care, since the current options were predicted to have no efficacy.

Finally, Group 4 made up 59.3% of all cases, demonstrating the relatively high frequency in which the cResponse test uncovered efficacious treatment options when the physician’s first choice was predicted to be ineffective. This outcome is similar to the result on the previously analyzed 45 patients with clinical outcomes, where 64.4% of patients (29/45) were shown to have clinically superior scores based on the test. This larger cohort further confirms the clinically validated results presented above, showing the high utility of the test in clinical practice.

Notably, we observed distinct patterns across the four defined groups based on cancer type (Supplementary Table S4). Group 1, representing cases where the physician’s planned treatment was the only effective option, showed a higher-than-average proportion in several cancers, including colon cancer, where this was the outcome in 25% of cases (7/28). In contrast, Group 2, where the physician’s choice was effective but alternative treatments were also identified, displayed a notable overrepresentation in bladder cancer, where this occurred in 52.9% of the cases (9/17). Most strikingly, Group 4, where the physician’s choice was ineffective, but the test identified at least one effective treatment, showed the potential importance of the test in cancers like breast cancer and pancreatic cancer, where it identified effective alternatives in 65.9% (62/94) and 60% (9/15) of the cases, respectively. These findings underscore the test’s value in personalizing cancer therapy and improving outcomes, particularly in cases where traditional approaches are less effective in advanced lines of therapy.

## 4. Discussion

The results of this study demonstrate the application of the cResponse test in real world oncology care. By focusing on advanced stage patients, we showed that 59.3% of the patients who used our test were predicted to be non-responders based on the physician’s first-choice treatment and were able to identify an alternative effective therapeutic option using the platform. In our cohort of patients whose clinical outcomes were reported and would have failed in response to the physician’s treatment based on cScore, we found that oncologists made use of the test 51.1% of the time, yielding a 91.18% PPV, a number significantly higher than response rates in routine advanced cancer treatments [14,15,16]. These data suggest that the cResponse test has the ability to uncover more effective treatments than the physicians’ choice alone.

These findings highlight the potential value of integrating the cResponse test into routine practice. Importantly, the results confirm the tests’ previously published sensitivity, specificity, PPV and NPV. In contrast, previous functional precision medicine tests have been developed to predict cancer treatment response, yet their clinical applicability remains questionable. Patient-derived xenograft (PDX) models, while providing an in vivo environment for tumor growth, require extended time (often months) and have shown response concordance rates of approximately 60–80%, limiting their ability to provide timely clinical recommendations [17]. In a study by Hidalgo et al., only 56% of PDX models successfully engrafted, and clinical translation was hindered by the absence of human immune components. Organoid-based platforms, which culture tumor cells in 3D environments, have demonstrated predictive accuracies ranging from 65 to 85%**,** but they can yield low success rates in growing tissue from aggressive tumors (30–50%) and may require weeks to generate treatment results [18,19].

Nonetheless, the current study has several important limitations. Since our data were received from post-market surveillance, we only report 16.1% (45/278) of the clinical data points, a relatively small number of patients. While the majority of these 45 were from advanced lines (third line and on) and comprised 10 different cancer types, reporting populations may have certain biases versus the non-reporting group and should not be considered representative of the entire 312 patients. Notably, the clinical accuracy from this study corresponds with our previous clinical trial showing a sensitivity of 96% and a specificity of 77.7% [12], suggesting congruity across the investigations. Lastly, since this was a post-market surveillance study, the accuracy was determined using patient reported data, which was obtained from their medical history, either based on a radiological evaluation or biochemical outcomes and was not confirmed by RECIST analysis.

The results of the test may have implications for treatment in multiple clinical situations. For patients, the ability to transform non-responders into responders means increased chances of disease control and prolonged survival. For oncologists, the test provides a possibly useful decision-support tool that enables more precise and personalized treatment planning, reducing the trial-and-error approach often associated with cancer care in advanced lines of therapy. Additionally, by identifying multiple effective treatments in 18.3% of cases (Group 2), the test could offer flexibility in therapy selection, allowing for considerations such as toxicity reduction, cost optimization, and treatment sequencing. These possibilities may have an impact on health economics and structuring payer responsibilities in future clinical practice.

While the clinical utility was demonstrated by oncologists selecting a potentially superior treatment shown on the cResponse test, we find that alternative cResponse outcomes can also yield benefits to the oncologist and patient. Whether by confirming the efficacy of the physician’s choice (Group 1), offering additional viable treatments (Group 2), ruling out ineffective options (Group 3), or identifying an effective alternative treatment (Group 4), the test has the potential to provide useful and applicable clinical information. This approach increases the possibility that a patient will receive benefit from the test even if a superior treatment is not identified.

According to previously reported data, most patients do not benefit clinically from genomic tests, which are considered the standard of care [2,20]. The cResponse test could be applied in addition to these genomic tests and possibly improve treatment selection and patient outcomes. This additional level of utility may support oncologists with tools to make evidence-based decisions. Recently, several reports have published genomic based tests that can predict whether a patient will respond to immunotherapy [21,22]. While these tests are beneficial for avoiding ineffective treatment, they do not suggest possible alternatives, leaving lower utility for the treating oncologist than tests that also suggest new treatment possibilities.

In the future, the cResponse test may also provide a suggested treatment list to the oncologist prior to evaluation, based on their patient’s sample. As more cResponse tests are performed and results recorded, there is an expanding database of therapeutics that are identified as potentially effective for specific tumor type profiles. Presenting these data to the oncologist as they select the drugs for evaluation may further improve the patient’s outcome and provide support assistance for the physician to maximize the benefits of the test.

## 5. Conclusions

In summary, the current study presents real world evidence that the cResponse test can address clinical challenges by aiming to improve response rates while supporting the goals of value-based oncology care. By optimizing resource utilization, minimizing potential patient harm, and providing actionable insights, the test may contribute to the ongoing efforts in advancing personalized cancer treatment. Further research and integration of the cResponse platform into clinical practice is needed to uncover its potential to enhance oncology care. Specifically, a pivotal blinded and randomized interventional clinical study applying the cResponse test in a prospective evaluation compared to a control arm of physicians’ care is necessary to validate the efficacy and utility of the platform. This future clinical study could also include side by side comparison to patient-derived organoids or CTC-based tests, to further benchmark the clinical performance of cResponse with existing functional assays. The preparation for this study is underway at clinical sites and will be the subject of a future paper outlining the design and initial results.

## Figures and Tables

**Table 1:** Clinical performance of cResponse. After receiving the cResponse report, patients and oncologist self-reported the outcomes of their treatments. Their responses were compared with cResponse predictions and calculated for accuracy.

Clinical Performance	Outcome	Confidence Interval
Sensitivity	96.88% (31/32)	0.83–0.99
Specificity	76.92% (10/13)	0.46–0.94
PPV	91.18% (31/34)	0.79–0.96
NPV	90.91% (10/11)	0.58–0.98

**Table 2:** Distribution of clinical cases based on cResponse predicted outcomes. cResponse results were divided into four categories based on outcomes showing the opportunity for clinical actionability. Most patients were predicted to not respond to the physician’s first choice but were found to be responders to additional treatment under consideration.

Category	Description	Percent of Cases
Group 1	Physician’s choice is the only effective choice	6.1%
Group 2	Physician’s choice and additional therapies equally effective	18.3%
Group 3	All therapies tested show no efficacy	16.1%
Group 4	Physician’s choice shown to be ineffective while additional drugs shown to yield response	59.3%

## Data Availability

The datasets presented in this article are not all readily available due to privacy and ethical restrictions. Requests to access the data should be directed to vered@curesponse.tech.

## Supplementary Materials

The following supporting information can be downloaded at: https://www.mdpi.com/article/10.3390/jpm16060298/s1, Table S1: Patients Included in the Analysis with Known Clinical Data. 30 patients reported on the outcomes of the treatments recommended by the cResponse assay. Their clinical profiles and response are listed here; Table S2: cResponse Recommendations Which Outperformed Physician’s Intent to Treat. 29 patients had cResponse scores that showed the physician’s intent to treat drug would yield non-response (a score less than 45) while one of the additional drugs selected for evaluation yielded a score indicative of a potential response (a score greater than 45). Patient numbers marked in red are the 6 instances where the physician did not follow the cResponse recommended choice and made use of the intent to treat decision despite the low score; Table S3: Distribution of Cancer Types Among Tests Performed. Patients provided their gender, age and cancer type when their cancer sample was submitted for analyiss. 278 cancer reports were then generated and the patient characteristics, as well as the percentage of each cancer sub-group type are presented; Table S4: Distribution of Cancer Type & Corresponding Group. Based on our classification of Groups 1-4, here we present the distribution of each response type in its relevant cancer sub-group type.

## References

[1] Majumder B., Baraneedharan U., Thiyagarajan S., Radhakrishnan P., Narasimhan H., Dhandapani M., Brijwani N., Pinto D.D., Prasath A., Shanthappa B.U. (2015). Predicting clinical response to anticancer drugs using an ex vivo platform that captures tumour heterogeneity. Nat. Commun..

[2] Le Tourneau C., Kurzrock R. (2016). Targeted therapies: What have we learned from SHIVA?. Nat. Rev. Clin. Oncol..

[3] Colomer R., Miranda J., Romero-Laorden N., Hornedo J., González-Cortijo L., Mouron S., Bueno M.J., Mondéjar R., Quintela-Fandino M. (2023). Usefulness and real-world outcomes of next generation sequencing testing in patients with cancer: An observational study on the impact of selection based on clinical judgement. eClinicalMedicine.

[4] Friedman A.A., Letai A., Fisher D.E., Flaherty K.T. (2015). Precision medicine for cancer with next-generation functional diagnostics. Nat. Rev. Cancer.

[5] Meijer T.G., Naipal K.A., Jager A., van Gent D.C. (2017). *Ex Vivo* tumor culture systems for functional drug testing and therapy response prediction. Future Sci. OA.

[6] van den Tempel N., Naipal K.A.T., Raams A., van Gent D.C., Franckena M., Boormans J.L., Kanaar R. (2018). Ex vivo assays to predict enhanced chemosensitization by hyperthermia in urothelial cancer of the bladder. PLoS ONE.

[7] Larsson P., Engqvist H., Biermann J., Werner Rönnerman E., Forssell-Aronsson E., Kovács A., Karlsson P., Helou K., Parris T.Z. (2020). Optimization of cell viability assays to improve replicability and reproducibility of cancer drug sensitivity screens. Sci. Rep..

[8] Joyce J.A. (2005). Therapeutic targeting of the tumor microenvironment. Cancer Cell.

[9] Gavert N., Zwang Y., Weiser R., Greenberg O., Halperin S., Jacobi O., Mallel G., Sandler O., Berger A.J., Stossel E. (2022). Ex vivo organotypic cultures for synergistic therapy prioritization identify patient-specific responses to combined MEK and Src inhibition in colorectal cancer. Nat. Cancer.

[10] Grinshpun A., Gavert N., Granit R.Z., Masuri H., Ben-Porath I., Breuer S., Zick A., Rosenberg S., Maoz M., Granit A. (2018). Ev vivo organ culture as potential prioritization tool for breast cancer targeted therapy. Cancer Biol. Ther..

[11] Ben-Hamo R., Jacob Berger A., Gavert N., Miller M., Pines G., Oren R., Pikarsky E., Benes C.H., Neuman T., Zwang Y. (2020). Predicting and affecting response to cancer therapy based on pathway-level biomarkers. Nat. Commun..

[12] Golan S., Bar V., Salpeter S.J., Neev G., Creiderman G., Kedar D., Aharon S., Turovsky L., Zundelevich A., Shahar H. (2023). A clinical evaluation of an ex vivo organ culture system to predict patient response to cancer therapy. Front. Med..

[13] Bhamidipati D., Verma A., Sui D., Maru D., Mathew G., Lang W., Posadas J., Hein J., Kopetz S., Futreal A. (2021). An analysis of research biopsy core variability from over 5000 prospectively collected core samples. npj Precis. Oncol..

[14] Olson D.J., Eroglu Z., Brockstein B., Poklepovic A.S., Bajaj M., Babu S., Hallmeyer S., Velasco M., Lutzky J., Higgs E. (2021). Pembrolizumab Plus Ipilimumab Following Anti-PD-1/L1 Failure in Melanoma. J. Clin. Oncol..

[15] Maldonado E.B., Parsons S., Chen E.Y., Haslam A., Prasad V. (2020). Estimation of US patients with cancer who may respond to cytotoxic chemotherapy. Future Sci. OA.

[16] Chen E.Y., Raghunathan V., Prasad V. (2019). An Overview of Cancer Drugs Approved by the US Food and Drug Administration Based on the Surrogate End Point of Response Rate. JAMA Intern. Med..

[17] Hidalgo M., Bruckheimer E., Rajeshkumar N.V., Garrido-Laguna I., De Oliveira E., Rubio-Viqueira B., Strawn S., Wick M.J., Martell J., Sidransky D. (2011). A pilot clinical study of treatment guided by personalized tumorgrafts in patients with advanced cancer. Mol. Cancer Ther..

[18] Pauli C., Hopkins B.D., Prandi D., Shaw R., Fedrizzi T., Sboner A., Sailer V., Augello M., Puca L., Rosati R. (2017). Personalized *In Vitro* and *In Vivo* Cancer Models to Guide Precision Medicine. Cancer Discov..

[19] Vlachogiannis G., Hedayat S., Vatsiou A., Jamin Y., Fernández-Mateos J., Khan K., Lampis A., Eason K., Huntingford I., Burke R. (2018). Patient-derived organoids model treatment response of metastatic gastrointestinal cancers. Science.

[20] Le Tourneau C., Delord J.P., Gonçalves A., Gavoille C., Dubot C., Isambert N., Campone M., Trédan O., Massiani M.A., Mauborgne C. (2015). Molecularly targeted therapy based on tumour molecular profiling versus conventional therapy for advanced cancer (SHIVA): A multicentre, open-label, proof-of-concept, randomised, controlled phase 2 trial. Lancet Oncol..

[21] Aggarwal C., Ben-Shachar R., Gao Y., Hyun S.W., Rivers Z., Epstein C., Kaneva K., Sangli C., Nimeiri H., Patel J. (2023). Assessment of Tumor Mutational Burden and Outcomes in Patients with Diverse Advanced Cancers Treated with Immunotherapy. JAMA Netw. Open.

[22] Arnon J., Dinstag G., Tirosh O., Gugel L., Kinar Y., Gottlieb T., Elia A., Rottenberg Y., Nechushtan H., Tabi M. (2025). Predictive value of ENLIGHT-DP in patients with metastatic lung adenocarcinoma treated with immune checkpoint inhibitors and platinum chemotherapy directly from histopathology slides using inferred transcriptomics. J. Immunother. Cancer.

