# Supplementary material for: Clinical Utility of an Ex Vivo Functional Test in Personalized Cancer Treatment
Source: J Pers Med. 2026 May 31;16(6):298. doi: 10.3390/jpm16060298 (PMC13301710; doi:10.3390/jpm16060298)
Supplement: Supplementary file 1 [file jpm-16-00298-s001.zip › jpm-4258722-supplementary.pdf]

Supplementary Table S1

Patient	Age	Gender	Cancer Type	Follow Up	Response	Treatment Line
99210621003	7	Male	GBM	No-Response	TN	NA
99160122019	47	Female	Breast	Response	TP	4
9914032022029	45	Female	Breast	Response	TP	2
9907042022036	43	Male	Pancreas	Response	TP	2
9921062022051	40	Female	Breast	Response	TP	1
9909082022064	55	Female	Colon	Response	TP	1
9925102022078	36	Male	Unknown	No-Response	TN	4
9909112022084	57	Female	Lung	Response	TP	3
9923012023114	54	Female	Breast	Response	TP	3
9902022023119	61	Female	Lung	No-Response	FP	2
9914032023134	61	Female	Colon	Response	TP	2
9927032023140	58	Female	Sarcoma	No-Response	TN	Unknown
9929032023142	47	Male	Pancreas	Response	TP	4
9929032023143	52	Female	Breast	No-Response	TN	4
19904042023150	44	Female	Breast	No-Response	TN	4
19924042023160	53	Female	Breast	Response	TP	5
19931072023190	48	Female	Breast	No-Response	TN	4
19908082023194	62	Female	Breast	No-Response	FP	7
19915082023198	50	Male	Mesothelioma	No-Response	TN	Unknown
19930082023205	41	Female	Renal	Response	TP	2
19918092023212	48	Female	Breast	Response	TP	6
19910102023222	17	Male	Sarcoma	No-Response	TN	3
19902112023229	76	Male	Renal	Response	TP	5
19914112023231	52	Female	Breast	Response	TP	4
19905122023234	51	Male	Unknown	No-Response	TN	7
19911012024245	67	Female	Breast	Response	TP	6
19922012024249	44	Female	Breast	No-Response	FP	5
19903062024295	41	Male	Colon	Response	TP	4
19903042024273	38	Female	Breast	Response	TP	2
19925062023178	46	Female	Breast	Response	TP	6
19914112024331	19	Female	Colon	Response	TP	2
19905062024296	76	Male	Pancreas	Response	FN	1
19927052024291	66	Male	Pancreas	Response	TP	2
19924042024280	64	Female	Breast	Response	TP	3
19908042024275	48	Female	Breast	Response	TP	6
19902012024241	78	Female	Breast	Response	TP	Unknown
19901052024002	72	Female	Lung	Response	TP	2
19908072024303	51	Male	Renal	Response	TP	4
19919032025365	5	Male	Sarcoma	Response	TP	2
19903032025358	19	Female	Sarcoma	Response	TP	2
19918102023223	60	Female	Lung	Response	TP	4
19917072024306	69	Female	Lung	Response	TP	4
19904032025360	2	Male	Neuroblastoma	No-Response	TN	Unknown
19911112024005	17	Female	RCC	Response	TP	3
9923012023116	54	Female	Breast	Response	TP	4

Supplementary Table S1. Patients Included in the Analysis with Known Clinical Data. 30 patients reported on the outcomes of the treatments recommended by the cResponse assay. Their clinical profiles and response are listed here.

Supplementary Table S2

Patient	Cancer Type	Group	Physician First choice	cScore	Treatment Based on cResponse	cScore
99160122019	Breast	4	Fulvestrant+Alpelisib	33	Doxorubicin	50
9925102022078	Unknown	4	Abemaciclib+Trametinib	11	Sorafenib	49
9909112022084	Lung	4	Cisplatin+Lenvatinib	21	Ifosfamide + Etoposide	71
9923012023114	Breast	4	5FU	9	Lapatinib	55
9902022023119	Lung	4	Cisplatin+Etoposide	28	Docetaxel	66
9929032023142	Pancreas	4	5FU	26	Gemzar+Oxaliplatin	63
9929032023143	Breast	4	Enhertu	15	5Fu	44
19904042023150	Breast	4	Taxol	26	Eribulin	48
19924042023160	Breast	4	Doxorubicin	34	Eribulin	59
19931072023190	Breast	4	CMF	22	Yondelis	46
19908082023194	Breast	4	Taxol+avastin	17	Vincristine	63
19930082023205	Renal	4	Cabozantinib	22	Ipi+Nivo	50
19918092023212	Breast	4	Enhertu	39	Trodelvy	71
19902112023229	Renal	4	Ipilimumab+Nivolumab	13	Cabozantinib+Lenvima	67
19914112023231	Breast	4	Enhertu	32	Taxol	49
19905122023234	Unknown	4	Ipilimumab+Nivolumab	13	FOLFOX+Cetuximab+Selumetinib	59
19903062024295	Colon	4	Ipilimumab+Nivolumab	39	Ribociclib+Trametinib	80
19925062023178	Breast	4	Enhertu	26	Carbo+Gemzar (+Keytruda)	61
19914112024331	Colon	4	FOLFOX	18	5FU+Oxaliplatin+Cetuximab	47
19905062024296	Pancreas	4	FOLFIRINOX	20	Gemzar+Abraxane	60
19927052024291	Pancreas	4	Cisplatin+Gemzar	30	Gemzar+Abraxane	50
19924042024280	Breast	4	5FU+Avastin	43	Carbo+Gemzar	63
19908042024275	Breast	4	Navelbine	38	Eribulin	67
19901052024002	Lung	4	Afatinib	10	Alimta	60
19908072024303	Renal	4	5FU+Gemzar	17	Carboplatin+Alimta	66
19903032025358	Sarcoma	4	Ifosfamide	6	Ifosfamide+Etoposide+Regorafenib	56
19918102023223	Lung	4	Alimta	15	Amivantamab	67
19911112024005	RCC	4	Olaparib	15	Everolimus	52
9923012023116	Breast	4	Doxorubicin	18	Taxol	98

Supplementary Table S2. cResponse Recommendations Which Outperformed Physician's Intent to Treat. 29 patients had cResponse scores that showed the physician's intent to treat drug would yield non-response (a score less than 45) while one of the additional drugs selected for evaluation yielded a score indicative of a potential response (a score greater than 45). Patient numbers marked in red are the 6 instances where the physician did not follow the cResponse recommended choice and made use of the intent to treat decision despite the low score.

Supplementary Table S3

Number of patients		278

Patient characteristic			
Age in years range (median)		2-91 (46.5)	
Male Gender - n		93	
Female Gender - n		176	
NA		9	

Cancer characteristics		n	
Cancer type -n(%)	Adenocortical	4	1.44%
	Breast	94	33.81%
	Bladder	17	6.12%
	Cervix	4	1.44%
	CRC	28	10.07%
	Endometrium	3	1.08%
	Esophagus	2	0.72%
	GBM	2	0.72%
	Head&Neck	4	1.44%
	Lung	21	7.55%
	Meningioma	2	0.72%
	Mesothelioma	2	0.72%
	Ovarian	17	6.12%
	Pancreas	15	5.40%
	Prostate	8	2.88%
	Renal	6	2.16%
	Sarcoma	21	7.55%
	Thymoma	2	0.72%
	Other	19	6.83%
	Unknown	7	2.52%
	Total	278	100.00%

Supplementary Table S3. Distribution of Cancer Types Among Tests Performed. Patients provided their gender, age and cancer type when their cancer sample was submitted for analyiss. 278 cancer reports were then generated and the patient characteristics, as well as the percentage of each cancer sub-group type are presented.

Supplementary Table S4

Cancer Tissue Type	# Patients	1	2	3	4
Adrenocortical	4		1	1	2
Bladder	17	2	9	4	2
Brain - Glioblastoma	2	1		1	
Brain - Meningioma	2		1	1	
Breast	94	3	20	9	62
Cervix	4			1	3
Colon	28	7	5	8	8
Endometium	3	1			2
Esophagus	2		1		1
Head and Neck	4			1	3
Lung	21		3	2	16
Mesothelioma	2			1	1
Ovarian	17	2	3	1	11
Pancreas	15	1	3	2	9
Prostate	8			1	7
Renal	6			1	5
Sarcoma	21		3	4	14
Thymoma	2				2
Other	19		3	5	11
Unknown	7			1	6
Total	278	17	52	44	165

Supplementary Table S4. Distribution of Cancer Type & Corresponding Group. Based on our classification of Groups 1-4, here we present the distribution of each response type in its relevant cancer sub-group type.
